# Ectopic Telomerase Expression Fails to Maintain Chondrogenic Capacity in Three-Dimensional Cultures of Clinically Relevant Cell Types

**DOI:** 10.1089/biores.2018.0008

**Published:** 2018-02-01

**Authors:** Tina P. Dale, Nicholas R. Forsyth

**Affiliations:** Faculty of Medicine and Health Sciences, Guy Hilton Research Center, Institute for Science and Technology in Medicine, Keele University, Staffordshire, United Kingdom.

**Keywords:** cartilage, chondrocyte, chondrogenesis, MSC, sulfated glycosaminoglycans, telomerase

## Abstract

The poor healing capacity of cartilage and lack of effective treatment for associated disease and trauma makes it a strong candidate for a regenerative medicine approach. Potential therapies tested to date, although effective, have met with a number of intrinsic difficulties possibly related to limited autologous chondrocyte cell yield and quality of cartilage produced. A potential mechanism to bypass limited cell yields and improve quality of differentiation is to immortalize relevant cell types through the ectopic expression of telomerase. Pellet cultures of human chondrocytes (OK3), bone marrow mesenchymal stem cells (BMA13), and embryonic stem cell (H1 line)-derived cells (1C6) and their human telomerase reverse transcriptase (*hTERT*) transduced counterparts were maintained for 20 days in standard maintenance medium (MM) or transforming growth factor-β3-supplemented prochondrogenic medium (PChM). Pellets were assessed for volume and density by microcomputed tomography. Quantitative gene expression (*COL1A2*, *COL2A1*, *COL3A1*, *COL6A3*, *COL10A1*, *ACAN*, *COMP*, *SOX9*); sulfated glycosaminoglycans (sGAGs), and DNA quantification were performed. Histology and immunohistochemistry were used to determine matrix constituent distribution. Pellet culture in PChM resulted in significantly larger pellets with an overall increased density when compared with MM culture. Gene expression analysis revealed similarities in expression patterns between telomerase-transduced and parental cells in both MM and PChM. Of the three parental cell types examined OK3 and BMA13 produced similar amounts of pellet-associated sGAG in PChM (4.62 ± 1.20 and 4.91 ± 1.37 μg, respectively) with lower amounts in 1C6 (2.89 ± 0.52 μg), corresponding to 3.1, 2.3, and 1.6-fold increases from day 0. In comparison, telomerase-transduced cells all had much lower sGAG with OK3H at 2.74 ± 0.11 μg, BMA13H 1.29 ± 0.34 μg, and 1C6H 0.52 ± 0.01 μg corresponding to 1.2, 0.87, and 0.34-fold changes compared with day 0. Histology of day 20 pellets displayed reduced staining overall for collagens and sGAG in telomerase-transduced cells, most notably with alterations in aggrecan and collagen VI; all cells stained positively for collagen II. We conclude that while telomerase transduction may be an effective technique to extend cellular proliferative capacity, it is not sufficient in isolation to sustain a naive chondrogenic phenotype across multiple cell types.

## Introduction

In the event of tissue damage, or as a result of degenerative disease processes, mature articular cartilage fails to undergo adequate reparative processes as a result of its hypocellular, avascular nature.^1^ As a consequence, damage is persistent, with multiple joint tissues frequently undergoing considerable further degeneration culminating in osteoarthritis.^2^ Sufferers of osteoarthritis have a significantly reduced quality of life due to pain and loss of function, with a considerable concomitant social and economic burden.^3^ Treatment for osteoarthritis and similar joint disorders is generally restricted to pain management strategies until the damage is sufficiently extensive to warrant arthroplasty,^4^ although in many smaller joints this may not be possible.

The lack of adequate regenerative or reparative processes has long been described^5^; techniques associated with the regenerative medicine field, in particular the use of transplantation-based cell therapies, may offer hope for the treatment of cartilage defects, where current standard therapies are inadequate. To date, variants of the autologous chondrocyte implantation/transplantation procedure have been the most intensively studied and have resulted in improvements in repair, reductions in patient symptoms, and an improved quality of life.^6^ Nevertheless, closer examination of the cartilage repair site has shown that the tissue often does not achieve optimal regeneration to hyaline cartilage.^7,8^ The underlying causes of poor hyaline cartilage regeneration have yet to be elucidated and would be of great interest to the regenerative medicine community.

*In vitro* analysis of cellular differentiation processes can provide valuable information which may then inform the likely outcomes of prospective regenerative therapies. Unfortunately, the extensive study of primary cells, often used in such therapies, remains challenging due to the onset of replicative senescence in an aging cell population most probably as a result of progressive telomere shortening.^9–11^ This process is further exacerbated by changes in cell phenotype as a result of *in vitro* culture techniques; this is a particularly well-established issue with the dedifferentiation of primary chondrocytes^12^ and stress-induced senescence.^13^ One possible technique to intervene in critical telomere shortening and prevent phenotypic changes is the re-expression of the catalytic subunit of telomerase reverse transcriptase (human telomerase reverse transcriptase [hTERT]) to stabilize or extend shortened telomeres with the intended outcome of significantly extending the replicative lifespan of the cells, while simultaneously halting the phenotypic changes associated with cell aging.^14–17^

This study compared several metrics of chondrogenesis in pellet cultures of three human cell types (chondrocytes [OK3], mesenchymal stem cells [MSCs, BMA13], and H1 embryonic stem cell [ESC]-derived MSC-like cells [1C6]) with current or potential clinical application for cartilage cell-based therapies and their *hTERT*-transduced counterparts (OK3H, BMA13H, 1C6H). These cells have previously been examined during expansion and in a simple chondrogenic monolayer differentiation environment, where there were several changes in cell phenotype, including diminished chondrogenic capacity in comparison to the more naive parental cells.^18^ The present study utilized more biologically relevant three-dimensional (3D) pellet cultures to compare chondrogenesis across the three cell types and to determine whether this was sufficient to restore the chondrogenic capacity of the transduced cells. Using this technique we noted improved chondrogenesis in OK3H cells in pellets compared with monolayer; however, overall chondrogenic capacity remained diminished in the transduced cell populations in comparison to primary counterparts.

## Materials and Methods

### Cell culture

Primary human knee articular chondrocytes OK3 (PromoCell, 71-year-old female), human MSCs BMA13, H1 human embryonic stem cell (hESC)-derived MSC-like cells, and the corresponding *hTERT*-transduced cells OK3H, BMA13H, and 1C6H were previously isolated/transduced as described.^18,19^ To isolate BMA13 cells, commercially sourced bone marrow aspirate (Lonza) was seeded at a density of 1 × 10^5^ mononuclear cells/cm^2^ in tissue culture flasks precoated with 10 ng/mL fibronectin (Sigma) in phosphate-buffered saline (PBS). Cells were seeded in high-glucose Dulbecco's modified Eagle's medium (DMEM) (4.5 g/L glucose) supplemented with 5% (v/v) fetal bovine serum (FBS), 1% (v/v) l-Glutamine (l-Glut), 1% (v/v) nonessential amino acids (NEAA), and 1% (v/v) Penicillin/Streptomycin/Amphotericin B (Lonza); a 50% media change was performed after 7 days and a 100% media change at 14 days.^20^ BMA13 and OK3 underwent routine media changes twice weekly with maintenance media (MM) consisting of DMEM supplemented with 10% (v/v) FBS, 1% (v/v) l-Glut, 1% (v/v) NEAA, and 4 ng/mL basic fibroblast growth factor (bFGF; PeproTech).^21^ 1C6 were derived from the H1 ESC line as described by Forsyth and McWhir.^19^ Briefly, passage 72 H1 hESCs were plated in suspension culture to form embryoid bodies for 96 h before being disaggregated and plated on to gelatin-coated plates at clonal density. Clone 1C6 cells had confirmed expression of typical MSC markers and tripotent (osteogenic, chondrogenic, and adipogenic) differentiation. capacity^19^ Cells were cultured in a MM of knockout-DMEM (Life Technologies) supplemented with 10% (v/v) FBS, 1% (v/v) l-Glut, 1% (v/v) NEAA, 4 ng/mL bFGF, and 100 nM dexamethasone (Sigma). A complete media change was performed twice weekly. All cells were subcultured enzymatically at confluence using 0.25% trypsin/ethylenediaminetetraaceticacid (EDTA; Lonza). All cells and pellets were cultured in humidified, 2% oxygen, tri-gas-controlled incubators with 5% CO_2_ and 93% N_2_.

Transduction of cells with *hTERT* was achieved using the Phoenix-A (Amphotropic) system with a pBABE-hTERT construct. BMA13 were transduced at passage 2, OK3 at passage 4, and 1C6 at population doubling 36. Selection of transduced cells was performed with 1 mg/mL G418.^18^

### Chondrogenic differentiation

Pellets were created by centrifuging 2.5 × 10^5^ cells/pellet in 1 mL of media at 300 *g* for 3 min in 1.5-mL microcentrifuge tubes. Pellets were formed and cultured in their standard MM and in prochondrogenic medium (PChM) consisting of the cells' MM with reduced FBS (1% [v/v]), further supplemented with 10 ng/mL transforming growth factor (TGF)-β3 (PeproTech), 0.1 μM dexamethasone, 40 μg/mL l-proline (Sigma), 50 μM ascorbic acid phosphate (Sigma), 1% (v/v) insulin, transferrin, selenium (Sigma), and 1% (v/v) sodium pyruvate (Sigma). Pellets were cultured for 20 days with twice weekly media changes. Pellets were fixed and used at days 1 and 20 for microcomputed tomography (μCT) analysis and histology, and either frozen at −80°C, or processed immediately at days 0 and 20 for sulfated glycosaminoglycans (sGAGs), DNA, and gene expression quantification. Spent culture media were also processed for sGAG quantification.

### Microcomputed tomography

Pellets were fixed in 4% (w/v) paraformaldehyde overnight before being stored in PBS at 4°C. Immediately before scanning, samples were briefly blotted to remove excess PBS and scanned using a Scanco Medical μCT 40 scanner at 45 kVp/176 μA. For volume and density analysis, images were thresholded at a lower threshold value of 50 and the maximum upper threshold value of 1000.

### Pellet histology and immunohistochemistry

Pellets were fixed in 4% (w/v) paraformaldehyde overnight, serially dehydrated (70% [v/v] industrial methylated spirits, 90% [v/v] isopropanol, 100% [v/v] isopropanol, 100% [v/v] isopropanol), and embedded in paraffin wax. Blocks were sectioned at 10 μm, dewaxed and stained with Toluidine Blue (0.04% [w/v] Toluidine Blue in 0.2 M sodium acetate buffer), Picrosirius Red (0.1% [w/v] Picrosirius Red in saturated aqueous picric acid), and Hematoxylin and Eosin.

Sections for immunohistochemistry were dewaxed and antigen retrieval through heat-induced epitope retrieval in citrate buffer (pH 6) or enzymatic antigen retrieval (0.1% trypsin) was performed. Sections were blocked with 1% (w/v) bovine serum albumin for 1 h and incubated with the primary antibodies (collagen I, collagen II, collagen VI, collagen X, and aggrecan [Abcam]) overnight followed by processing with the appropriate secondary antibody ABC Immunoperoxidase Staining Kit (Santa Cruz Biotechnology).

### Dimethylmethylene blue sGAG and PicoGreen DNA quantification

For sGAG and DNA quantification, pellets and cumulative media were used at days 0 and 20. Pellets and media aliquots were digested by overnight incubation in 100 μL of 2.5 mg/mL proteinase K (Sigma) in 100 mM ammonium acetate (Sigma) at 57°C with periodic vortexing. Following digestion, 1 mL of ice-cold ethanol was added to each digest and samples left to precipitate overnight at −20°C. Samples were pelleted at 17,000 *g*, the ethanol was removed, and samples air dried before being resuspended in 100 mM ammonium acetate for analysis.

For sGAG quantification, 200 μL of dimethylmethylene blue (DMMB) solution (64.0 μg/mL DMMB, 3.0 mg/mL glycine, 2.4 mg/mL NaCl, all dissolved in 0.01 M HCl [all Sigma])^22^ was dispensed to the samples in 96-well microplates using an automated dispenser. Absorbance values were obtained immediately at 530 nm using a Synergy-2 plate reader (BioTek). Concentrations were determined using a bovine chondroitin sulfate (Sigma) concentration curve.

The Quant-iT PicoGreen double-stranded DNA assay (Life Technologies) was carried out according to the manufacturer's protocol. Duplicate sample aliquots were diluted 1 in 10 in Tris EDTA buffer in 96-well microplates before incubation with PicoGreen working solution in the dark. Plates were read using a Synergy-2 plate reader (excitation 480 nm, emission 520 nm). Concentrations were determined using a lambda DNA calibration curve.

### Gene expression analysis

Pellets were lysed and RNA extracted using the Qiagen RNeasy Mini Kit. Pellets were first physically disrupted using disposable, single-use pestles in the presence of a small amount of RLT lysis buffer supplemented with 10 μL/mL β-mercaptoethanol. To further disrupt proteinaceous material, 150 μL of 2.5 mg/mL proteinase K^23^ was added to the lysis buffer and samples incubated for 20 min at 57°C with vortexing. The resulting lysate was added to a QIAshredder and centrifuged at 17,000 *g* before the minispin column was discarded and the tube capped. All lysates were immediately processed for RNA extraction as per the manufacturer's protocol. Eluted RNA was quantified with a NanoDrop 2000 spectrophotometer (Thermo Scientific) and stored at −80°C until required.

Relative gene expression in cell pellets at day 0 and after 20 days in either MM or PChM was assessed using quantitative reverse transcription–polymerase chain reaction (qRT-PCR) with the SuperScript III Platinum SYBRGreen OneStep qPCR Kit (Life Technologies) and a Stratagene MX3005P real-time thermal cycler.

### Statistical analysis

Data are expressed as mean ± standard deviation unless otherwise indicated. qRT-PCR data were analyzed using REST-2009 with significance determined using a randomization algorithm.^24^ Hierarchical clustering was performed using the Hierarchical clustering algorithm in Gene Cluster 3.0^25^ using an uncentered correlation similarity metric and average linkage clustering with associated data viewed in Java TreeView.^26^ Statistical analysis of pellet size, DNA content, and sGAG production was by repeated measures two-way analysis of variance with Bonferroni post-tests of chondrogenic supplemented samples compared with nonsupplemented controls using GraphPad Prism 5.00 (GraphPad, San Diego, CA). Results were deemed to be statistically significant when *p* ≤ 0.05.

## Results

### Pellets could be formed in all conditions, but showed a strong reliance on a prochondrogenic environment for maintenance

**FIG. 1.**
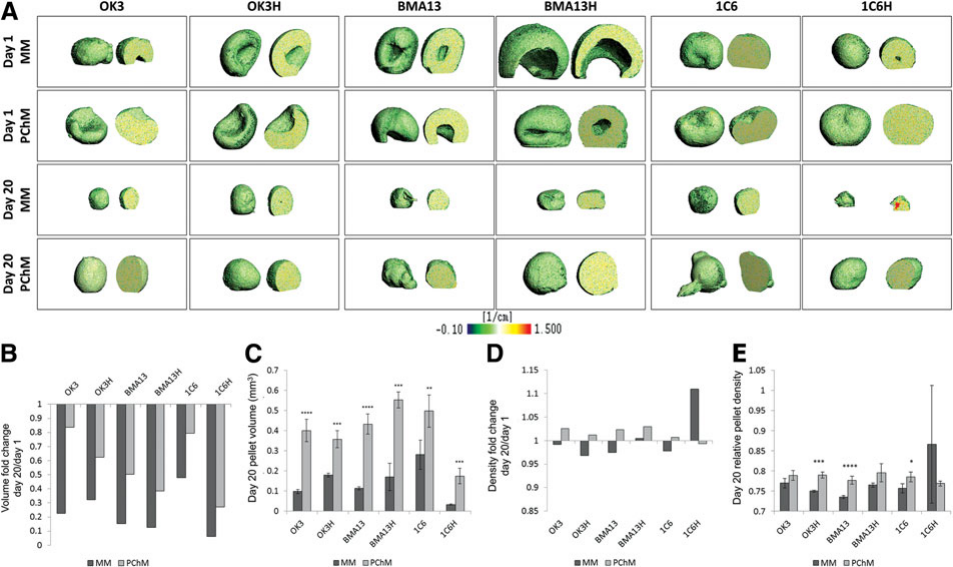
PChM supports stability and promotes increased pellet size. **(A)** μCT scans postfixation of day 1 and 20 pellets in MM or PChM indicate that pellets are formed by flattened, multilayered cell sheets folding or rolling to form spheroids. **(B)** Fold change in pellet volume relative to day 1. **(C)** Pellet volume at day 20. **(D)** Fold change in pellet density relative to day 1. **(E)** Pellet density at day 20. PD level for cells was as follows: OK3 PD6, OK3H PD50, BMA13 PD1, BMA13H PD16, 1C6 PD36, and 1C6H PD107. **p < 0.01, ***p < 0.001, ****p < 0.0001. Y-axis on **(B)** and **(D)** indicate fold change at day 20 relative to day 1, **(C)** pellet volume (mm^3^), **(E)** pellet density (relative units). Data are plotted as mean ± standard deviation, *n* = 3. μCT, microcomputed tomography; MM, maintenance medium; PChM, prochondrogenic medium; PD, population doubling.

Stable, free-floating pellets were formed in both MM and PChM within a 24-h period. μCT imaging of day 1 pellets (Fig. 1A) showed a process of folding or rolling of the multilayered, concave, sheet of cells formed by centrifugation, frequently resulting in a hollow core to the pellets. Over the 20-day time course early features caused by the folding process generally resolved with pellets having a more spheroidal, homogeneous appearance; early heterogeneity was more likely to persist in MM cultures.

The use of μCT enabled quantitative assessment of pellet volume and density. All pellets were found to be smaller at day 20 than day 1 (Fig. 1B). The decrease in size was always larger in MM cultures (0.061–0.479 × day 1) than in PChM (0.272–0.837 × day 1), such that by day 20 all pellets cultured in PChM were significantly larger than the corresponding MM cultures (Fig. 1C). In all cell types there was a smaller reduction in size in parental cell MM cultures compared with transduced MM cultures. With the exception of 1C6H pellets, culture in PChM also resulted in a trend for the formation of denser cell pellets (Fig. 1D) with this difference becoming significant in OK3H (*p* ≤ 0.001), BMA13 (*p* ≤ 0.0001), and 1C6 (*p* ≤ 0.05) (Fig. 1E).

### Pellet culture, media type, and *hTERT* transduction result in alterations in gene expression

**FIG. 2.**
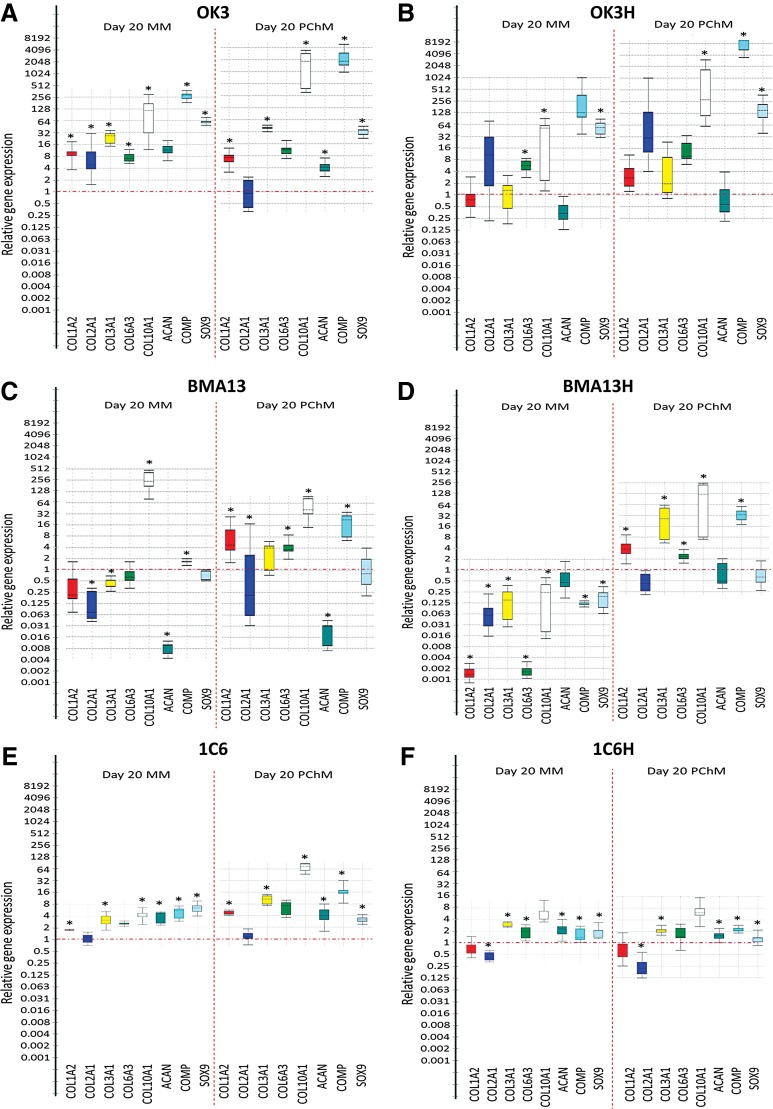
Pellet culture, media type, and *hTERT* transduction influence gene expression. Box and whisker plots indicating relative gene expression at day 20 determined using 2^−ΔΔCT^ with expression relative to the same-cell day 0 MM sample. **(A)** OK3 PD6. **(B)** OK3H PD50. **(C)** BMA13 PD1. **(D)** BMA13H PD18. **(E)** 1C6 PD36. **(F)** 1C6H PD107. *p ≤ 0.05. In all cases the Y-axis represents 2^−ΔΔCT^, the box represents the interquartile range, the whiskers the outer quartiles, and the line the median expression. hTERT, human telomerase reverse transcriptase.

Figure 2 shows relative fold change in gene expression in a panel of representative chondrogenic and hypertrophy/fibrocartilage markers (*COL1A2*, *COL2A1*, *COL3A1*, *COL6A3*, *COL10A1*, *ACAN*, *COMP*, and *SOX9*). Overall, although pellets displayed considerable heterogeneity in expression level, each cell type had similar patterns of gene expression in both MM and PChM, generally with a more muted response in MM, indicating a chondrogenic response induced by 3D culture in the absence of biochemical cues. Transduced population responses were similar overall to those of the parental cells. Chondrocytes and hESC-derived cells tended strongly toward upregulation in gene expression; with a single gene, *COL2A1*, significantly downregulated across all eight conditions (*p* ≤ 0.05). In contrast, MSCs, particularly BMA13H displayed a much stronger differential response to culture conditions with a greater trend for downregulation in MM (BMA13: 3/8 [*COL2A1*, *COL3A1*, *ACAN*] genes, BMA13H: 7/8 [*COL1A2*, *COL2A1*, *COL3A1*, *COL6A3*, *COL10A1*, *COMP*, *SOX9*]) genes, and upregulation in PChM (BMA13: 4/8 [*COL1A2*, *COL6A3*, *COL10A1*, *COMP*] genes, BMA13H: 5/8 [*COL1A2*, *COL3A1*, *COL6A3*, *COL10A1*, *COMP*] genes).

Interestingly, the transcription factor *SOX9*, considered the master regulator for chondrogenesis, was significantly (*p* ≤ 0.05) upregulated in OK3, OK3H, 1C6 and 1C6H in both media types at day 20 in contrast to the MSCs BMA13 and BMA13H where levels were not significantly different to day 0 cultures. Another of the paradigm markers for hyaline cartilage production *ACAN* was up-regulated in OK3, 1C6 and 1C6H pellets (*p* ≤ 0.05) while being strongly down-regulated in the primary MSCs BMA13 (*p* ≤ 0.05) and static in the transduced BMA13H pellets. *COL2A1* expression was up-regulated in OK3 MM cultures (*p* ≤ 0.05) but remained unchanged, relative to day 0, in OK3 PChM, OK3H MM and PChM, BMA13H PChM, 1C6 MM and PChM or was down-regulated in all other cultures (BMA13 MM and PChM, BMA13H MM, 1C6H MM and PChM) (*p* ≤ 0.05). Markers for fibrocartilage production and hypertrophy, *COL1A2* and *COL10A1*, were also frequently upregulated with *COL1A2* upregulation (*p* ≤ 0.05) in all parental cell pellet cultures with the exception of BMA13 MM. In contrast, transduced pellets were less likely to express *COL1A2* with significant upregulation noted in one transduced pellet condition only (BMA13H PChM). *COL10A1* was upregulated in all parental cell cultures, OK3H in both MM and PChM and BMA13H PChM (*p* ≤ 0.05). BMA13H MM samples displayed significant downregulation in line with the downregulation noted in all other genes with this sample.

**FIG. 3.**
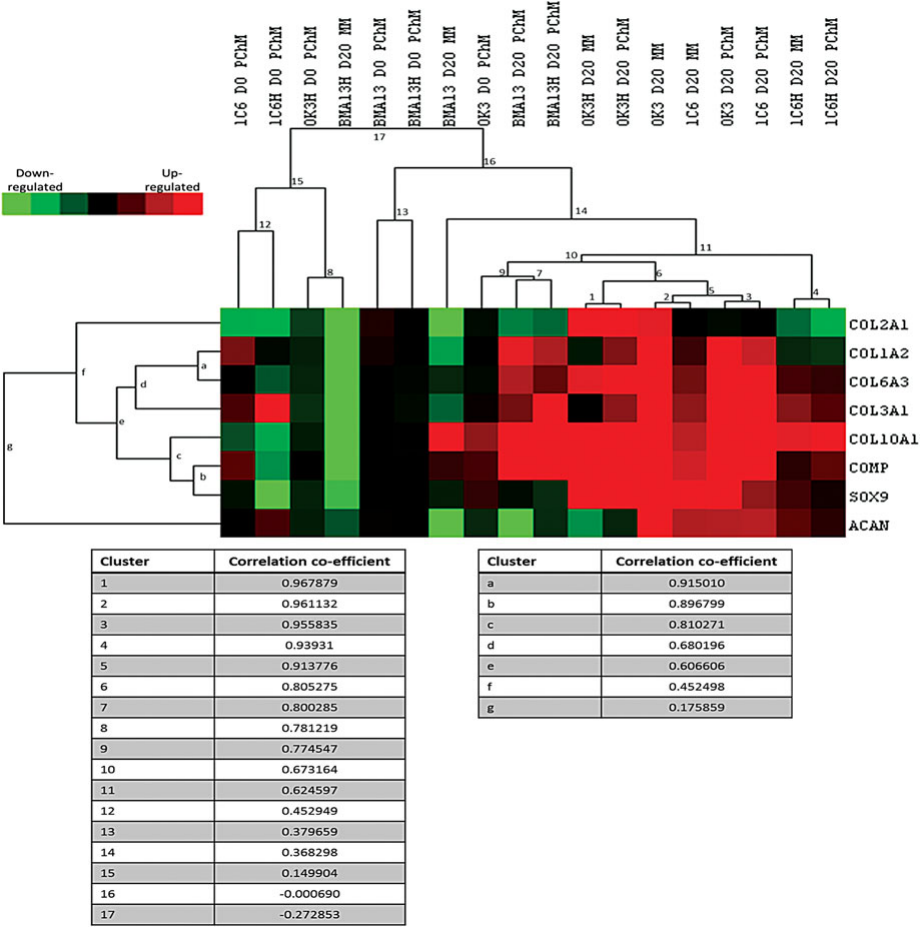
Hierarchical clustering highlights similarities in gene expression in MM and PChM and between chondrocytes and human embryonic stem cell-derived cells. Samples (excluding day 0 MM) were subjected to hierarchical clustering to identify sample group correlations in expression patterns. Red indicates upregulated genes, green downregulated genes, and black no change in expression. All expression changes are determined relative to the day 0 MM condition. PD level of cells is as follows: OK3 PD6, OK3H PD50, BMA13 PD1, BMA13H PD18, 1C6 PD36, and 1C6H PD107. Correlation coefficients for each cluster are listed below the heatmap.

Patterns of gene expression were explored quantitatively using a hierarchical clustering algorithm (Fig. 3). The strongest correlations in gene expression profiles were found with the chondrocytes and the hESC-derived cells (clusters 1–6, correlation coefficients [ρ] of >0.8). Primary chondrocytes OK3 clustered strongly with primary hESC-derived cells 1C6 in both MM (cluster 2, ρ 0.96) and PChM (cluster 3, ρ 0.96) conditions, whereas the transduced variant of these cell types OK3H (cluster 1, ρ 0.97) and 1C6H (cluster 4, ρ 0.94) clustered most strongly by cell type irrespective of media formulation. BMA13 and BMA13H showed similarities in expression in PChM (cluster 7, ρ 0.8). In MM, however, BMA13 clustered weakly with the other day 20 samples (cluster 14, ρ 0.37), whereas BMA13H clustered with day 0 OK3H PChM samples (cluster 8, ρ 0.78).

### *hTERT* transduction reduced sGAG production

**FIG. 4.**
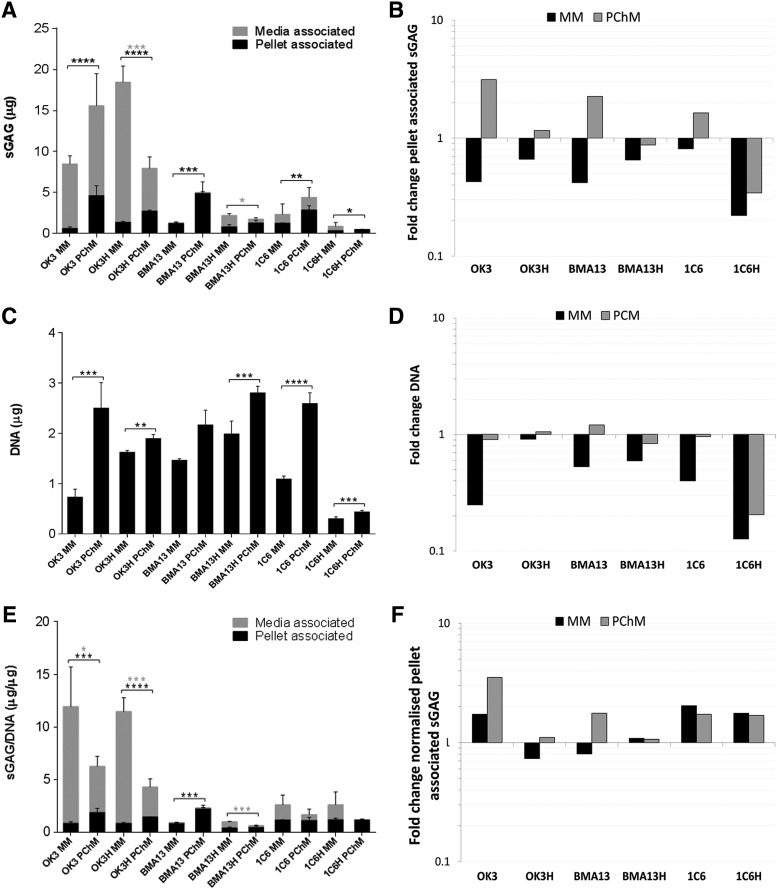
*hTERT* transduction reduced sGAG production compared with parental cells. sGAG and DNA content in pellets was quantified at day 0 and at day 20 after culture in MM or PChM for all cell pellets. **(A)** Absolute pellet- and media-associated sGAG at day 20. **(B)** Fold change in pellet-associated sGAG at day 20 relative to day 0. **(C)** Pellet DNA content at day 20. **(D)** Fold change in pellet DNA content at day 20 relative to day 0. **(E)** Pellet and media day 20 sGAG normalized to day 20 DNA content. **(F)** Fold change in normalized day 20 sGAG content relative to day 0 levels. PD level for cells was as follows: OK3 PD6, OK3H PD50, BMA13 PD6, BMA13H PD16, 1C6 PD36, and 1C6H PD86. *p ≤ 0.05, **p ≤ 0.01, ***p ≤ 0.001, ****p ≤ 0.0001. The Y-axes on **(A)** and **(C)** indicate sGAG and DNA quantity (μg) and **(B)** and **(D)** fold change in sGAG and DNA, respectively. The Y-axis on **(E)** represents sGAG normalized to DNA content (μg/μg) and on **(F)** fold change in normalized sGAG relative to day 0 levels. Data are plotted as mean ± standard deviation, *n* = 3. sGAG, sulfated glycosaminoglycan.

DMMB analysis showed higher sGAG content in pellets cultured in PChM than MM, achieving statistically significant levels in all but BMA13H pellets (Fig. 4A, B). Parental cell pellets cultured in PChM always contained more sGAG than their transduced counterparts (OK3 4.62 ± 1.20 μg, OK3H 2.74 ± 0.11 μg, BMA13 4.91 ± 1.37 μg, BMA13H 1.29 ± 0.34 μg, 1C6 2.89 ± 0.52 μg, 1C6H 0.52 ± 0.01 μg) as the transduced cells had only small gains, or losses, being particularly evident in 1C6H pellets, where sGAG levels decreased to only 0.22 and 0.34 × day 0 amounts in MM and PChM, respectively. Media sGAG levels were also determined and were found to be highest in chondrocyte-based cultures.

Pellet DNA content decreased in all MM cultures; PChM cultures generally maintained a DNA content much closer to day 0 levels resulting in pellets with more DNA following culture in PChM compared with MM, significantly so in all but BMA13. OK3H-transduced cells appeared to be particularly tolerant of MM culture, with much smaller reductions in DNA compared with the parental OK3 cells (0.91 vs. 0.25 × day 0). As with sGAG, reductions in DNA levels were particularly dramatic in 1C6H at only 0.13 and 0.21 × day 0 levels in MM and PChM.

Normalization of pellet sGAG to DNA maintained previously observed absolute sGAG trends across OK3, OK3H, BMA13, and BMA13H. In PChM BMA13 remained the highest sGAG producer at 2.23 ± 0.31 μg/μg followed by OK3 with 1.87 ± 0.41 μg/μg, and finally 1C6 with 1.13 ± 0.29 μg/μg. OK3H and BMA13H were reduced in comparison with 1.45 ± 0.03 μg/μg and 0.46 ± 0.14 μg/μg, respectively. These values remained significantly higher than those obtained for pellets cultured in MM with the difference greatest in BMA13 followed by OK3, and OK3H. BMA13H, 1C6, and 1C6H cell pellets cultured in PChM were not significantly different to MM cultures and most strikingly, normalized levels in 1C6H were similar to 1C6 levels at 1.20 ± 0.15 μg/μg and 1.16 ± 0.03 μg/μg and in MM, and 1.18 ± 0.08 μg/μg and 1.13 ± 0.29 μg/μg in PChM due to large losses in both sGAG and DNA as a result of pellet instability.

### *hTERT* transduction reduces overall collagen and sGAG extracellular matrix staining

**FIG. 5.**
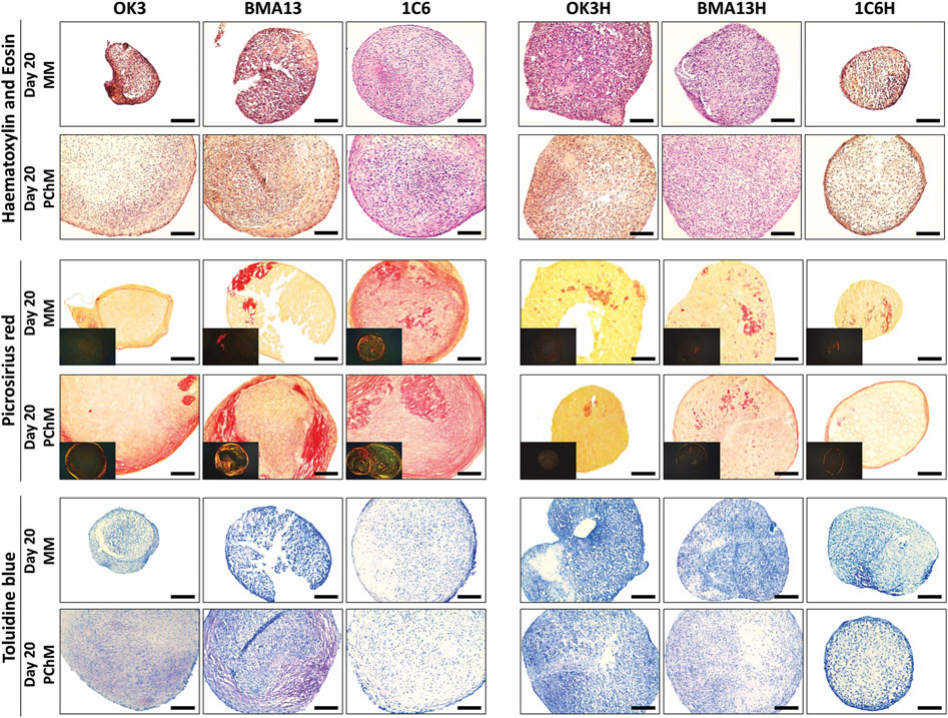
*hTERT* transduction reduced ECM staining for both sGAG and collagen. Histological staining of 10 μm-sectioned pellets at day 20 after culture in MM or PChM as indicated. Sections are stained with Hematoxylin and Eosin (upper); Picrosirius Red for collagen (middle); inset images are acquired under crossed polarizers; and Toluidine Blue (lower). PD level for cells was as follows: OK3 PD6, OK3H PD50, BMA13 PD6, BMA13H PD16, 1C6 PD36, and 1C6H PD86. PD level for cells was as follows: OK3 PD6, OK3H PD50, BMA13 PD6, BMA13H PD16, 1C6 PD36, and 1C6H PD86. Scale bar = 100 μm.

Histological analysis (Fig. 5) of parental cells showed more variability in staining between culture in MM and PChM than transduced cells. OK3 and BMA13 pellets cultured in MM had minimal intercellular staining for cartilaginous extracellular matrix (ECM) components; although OK3 appeared to develop a slightly more matrix-rich capsule, BMA13 pellets in MM were poorly formed and very friable upon sectioning. Notably, 1C6 pellets in MM, while being smaller than those cultured in PChM, were very similar in ECM content to PChM cultures. The culture of all parental cell pellets in PChM resulted in larger, more ECM-rich pellets with positive staining for both collagen and sGAG. Collagen fibers in OK3 pellets were finer and more homogeneously distributed, whereas BMA13 and 1C6 stained more intensely for collagen that was more heterogeneously distributed within the pellet. Crosspolarized images (inset) of BMA13 revealed fibers that were almost exclusively yellow to red, indicative of large, organized fibers^27^ with few of the finer green fibers that were present in 1C6 pellets. Toluidine Blue staining for sGAG followed a very similar pattern with intense metachromatic staining of sGAG in BMA13 pellets, corresponding with areas of high collagen deposition, while being more diffuse and homogeneous in OK3 and 1C6 pellets. Transduced cells, OK3H and BMA13H, behaved dissimilarly to parental counterparts, forming larger more stable pellets; however, only minimal matrix staining was observed.

### *hTERT* transduction reduced collagen VI and aggrecan incorporation into the ECM

**FIG. 6.**
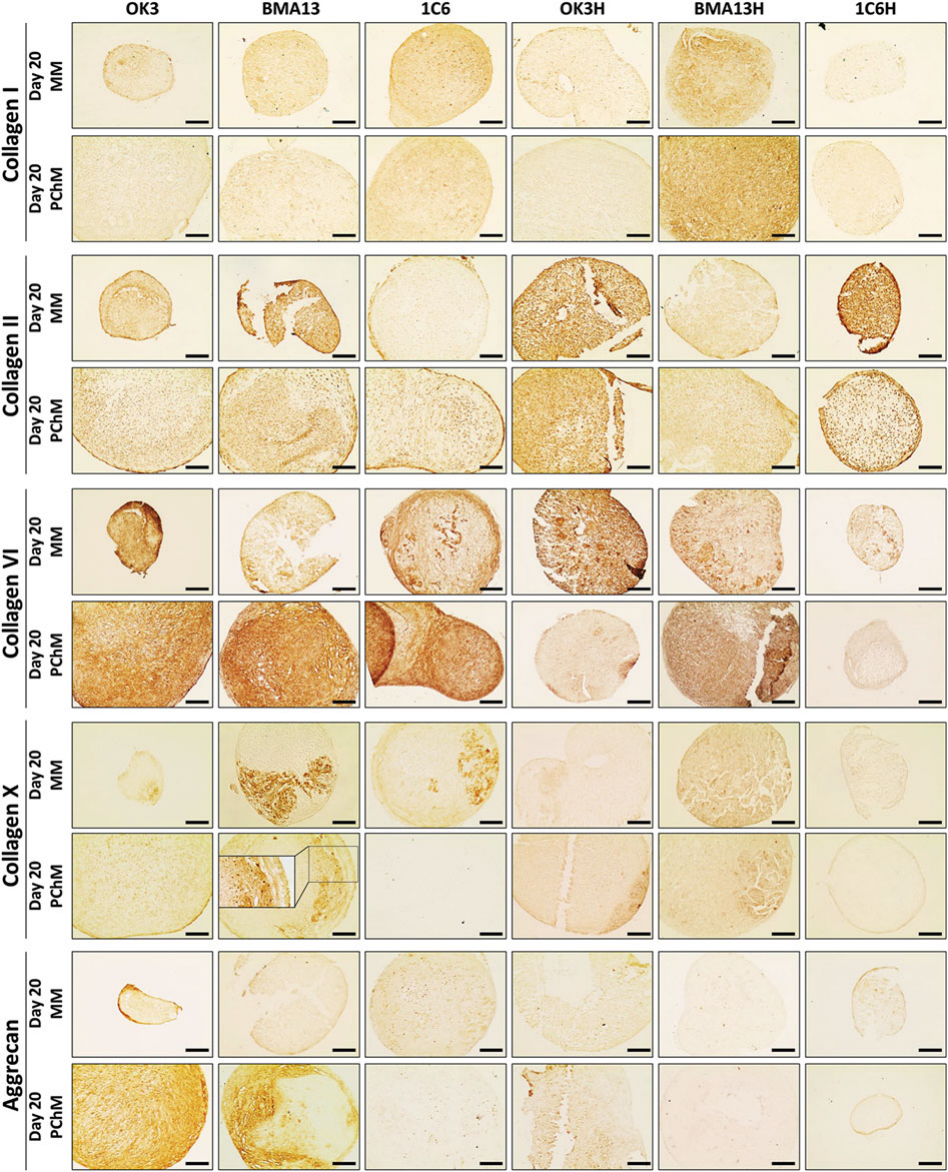
*hTERT* transduction reduced collagen VI and aggrecan incorporation into the ECM. Immunoperoxidase staining of 10 μm-sectioned pellets at day 20 after culture in MM or PChM as indicated. Sections were stained for collagen types I, II, VI, and X, and aggrecan. PD level for cells was as follows: OK3 PD6, OK3H PD50, BMA13 PD6, BMA13H PD16, 1C6 PD36, and 1C6H PD86. Scale bar = 100 μm. ECM, extracellular matrix.

Immunoperoxidase staining (Fig. 6) for ECM proteins generally agreed with previous data; staining tended to be more pronounced in primary pellets and PChM than transduced counterparts and MM. The most prominent ECM molecules were collagen II, particularly pericellularly, and collagen VI; despite upregulation at the gene level only low levels of collagen I staining were detected. Marked aggrecan staining was restricted to OK3 cultures particularly in PChM and BMA13 in PChM, where staining was well defined and mirrored Toluidine Blue staining. Type X collagen was most evident in BMA13 MM pellets, where staining was regional, and to a lesser extent BMA13 PChM and 1C6 MM cultures; more subtle staining was evident in all other cultures.

## Discussion

Cell immortalization through the constitutive expression of hTERT to extend the proliferative potential of primary cells *in vitro* while maintaining a naive chondrogenic phenotype is an appealing prospect. We have previously shown that this approach can extend proliferative potential, including to the point of apparent immortality, but chondrogenic capacity is reduced during growth factor-induced monolayer chondrogenesis.^18^ We have now extended those observations using a pellet culture system to provide additional spatial differentiation cues that are believed to be beneficial to chondrogenesis,^28–30^ particularly in conjunction with reduced oxygen culture,^31,32^ to determine whether this was sufficient to ameliorate the loss in differentiation capacity previously seen. Despite introducing these modifications, we found that while all cells responded differentially to MM versus PChM, chondrogenesis was reduced in all *hTERT*-transduced cell populations.

All cells readily formed pellets and these were consistently larger with sustained DNA levels and increased sGAG after culture in PChM compared with MM. In the absence of biochemical prochondrogenic stimuli provided by PChM standard MM was generally unable to support long-term pellet stability, nor cell proliferation. This has significant treatment implications as transplantation of cells to an environment lacking adequate prochondrogenic stimuli is likely to lead to poor clinical outcomes. 1C6H performed particularly badly, most likely as a result of the transduction process, as the parental 1C6 cells formed stable pellets in PChM and in contrast to OK3 and BMA13, in MM. The 1C6 MM contains dexamethasone, which may be responsible for stabilizing the pellets and supporting a limited amount of chondrogenesis.^33^ This suggests that the administration of dexamethasone, either through the incorporation of drug-releasing scaffold or systemically, to patients receiving a cartilage cell therapy treatment may be beneficial; however, the results from preliminary animal studies are varied.^34,35^

Immortalized cell lines are generated to provide a long-term supply of cells with a stable phenotype for *in vitro* studies; however, the outcomes have been varied.^14,36–50^ The underlying variation in response has yet to be explained, however, a number of factors may be contributing. Studies examining variation in cell response at a single cell level using clonal populations have found that there are differences in outcome. This may be influenced by the variation in the starting cell population, for example MSCs are known to be a mixture of cells with varied potency^51,52^; similarly chondrocytes display differences in differentiation capacity,^53^ perhaps depending on their tissue zone of origin.^54^

The extensive expansion of cells, particularly in polyclonal populations, favors the selection of cells with high proliferative capacity. There is evidence that “cell competition”^55^ may lead to slowly proliferating clones arising during cell culture to be eliminated by apoptosis when in direct contact with faster growing cells.^56^ The concept that more proliferative cells are less likely to undergo differentiation has merit; the two processes of proliferation and differentiation are exclusive, with cells developing and exhibiting the features of a differentiated phenotype during interphase.^57^ Cells that spend less time in interphase due to more rapid cell cycling are therefore less likely to exhibit the features associated with a differentiated phenotype. The rapidly proliferating 1C6 cells and the more extensively expanded transduced cells all appear to have a less differentiated phenotype. One possibility to “rescue” cells that are more proliferative to potentially increase their differentiation capacity is the application of conditional or transient immortalization. Transient rather than constant expression of *hTERT* is sufficient to extend cellular lifespan and conditional or transient immortalization has been successfully used in several cell types, including fibroblasts,^58^ MSCs,^59^ beta cells,^60^ and chondrocytes.^61^

Interestingly overexpression of *hTERT* in the undifferentiated H1 hESC cell line, the parental line of 1C6, has independently been found to affect the differentiation capacity of the cells with reduced expression of germline markers and increased expression of the pluripotency marker *OCT4*.^37^ If 1C6 does not reflect a fully differentiated MSC-like cell, it is possible that the results herein represent a very similar response and explain why ectopic expression in 1C6H resulted in very poor differentiation. Despite the more muted chondrogenesis seen in 1C6 the sustained upregulation of genes associated with chondrogenesis, including *SOX9*, suggests that these cells may have a further capacity for chondrogenesis with additional differentiation cues. This is perhaps unsurprising when the complex routines employed for hESC chondrogenic differentiation^62,63^ are compared with the relatively simple PChM used in this study. An independent report on the chondrogenic capacity of another H1-derived, MSC-like cell type in comparison to MSCs yielded similar results to those herein. The response of the cells in that case was improved by the addition of BMP7 to cultures^64^ and this may be an option in future work.

In contrast to 1C6 and OK3 BMA13 had significant downregulation of the majority of chondrogenesis-associated genes, including *SOX9.* MSCs are prone to osteogenesis when implanted ectopically^65^ and there is no evidence as yet that they spontaneously form any other lineage-specific tissue type in the absence of the appropriate prodifferentiation stimulus.^66^ It has also been suggested that MSCs may experience a lag period before the onset of differentiation due to their stem cell state^67^ and extended culture has proved beneficial in some cases.^33,68^ Nevertheless, our results would suggest that this is not the case here as the MSCs progressed rapidly through a chondrogenic phase, where aggrecan was deposited to hypertrophy associated with downregulation of the *ACAN* and *SOX9* genes and upregulation of type I and type X collagen. These results are typical for both chondrocytes and MSCs and support much of the available literature.^33,69–71^ Further refinement of prochondrogenic culture conditions is ongoing.^71^ However, factorial screening of growth factor supplementation on an array of “wanted” and “unwanted” genes associated with chondrogenesis found no combination in which optimal gene expression for chondrogenesis of MSCs could be achieved, with both wanted and unwanted genes upregulated simultaneously; the study concluded that TGF-β1, 2, or 3 combined with dexamethasone supplementation still provided the overall best outcome.^72^ Alternative sources of MSCs, including those residing in the joint compartment, may provide cells more suited to chondrogenesis and there has been a recent interest in the application of synovial MSCs^73^ and cells from the infrapatellar fat pad^74^ as alternatives to bone marrow.

Biochemically, both the OK3 and BMA13 pellets in PChM were similar with respect to sGAG accumulation; however, while OK3 sGAG and collagen deposition was homogeneous, BMA13 evidenced a distinct regional pattern of sGAG staining that was mirrored by aggrecan and collagen deposition. Regional variations in matrix deposition have previously been attributed to biochemical gradients; however, the pattern of staining seen with BMA13 mirrors the folded pellet shape seen by μCT analysis. During pellet formation the cells on the periphery of centrifuged pellets are exposed to higher tension mediating changes in the cell cytoskeleton and morphology; these changes can induce significant downstream effects on cell behavior, including influencing cell differentiation. Higher stresses can influence MSCs such that they are directed more toward the osteogenic lineage.^75^ Studies where centrifuged cell pellets are compared with naturally aggregated micromasses report more optimal results in the micromasses indicating that the centrifugation technique impacts on the subsequent cell biology even within the same population.^76,77^ The overall result of this is that while biochemical cues remain important, cells are also receiving potentially competing cues as a result of mechanical forces and spatial patterning. The results herein suggest that MSCs may be more sensitive than the chondrocytes to these competing cues, probably due to their multipotent nature.

## Conclusion

In the presence of prochondrogenic cues, stable pellets undergoing chondrogenesis were formed from all parental cell types. However, in the absence of appropriate cues, pellets gradually diminished in size, DNA content, and the major chondrogenic ECM proteins. While chondrocytes and MSCs achieved biochemically equivalent sGAG content, there were notable differences in the spatial distributions of the ECM proteins resulting in heterogeneous, fibrous tissue formation in the MSC pellets, MSCs did not maintain *SOX9* expression and appeared to have progressed to a hypertrophic phenotype favoring the continued use of primary chondrocytes in cartilage cell therapy trials. Transduced cells had a reduced chondrogenic response compared with parental cells and in confirmation of previous two-dimensional (2D) results they do not represent a suitable replacement for primary cells. However, interestingly there were indications, such as the sustained differential response to MM and PChM, *SOX9* expression, and in particular the improvement seen by transferring OK3H from a 2D to a 3D environment, which suggested that the transduction while clearly significantly impacting upon the cells' chondrogenic potential had not completely abrogated the chondrogenic response, and future optimization of the immortalization process may produce cell lines with much greater *in vitro* utility.

## Abbreviations Used

μCT: microcomputed tomography
2D: two-dimensional
3D: three-dimensional
bFGF: basic fibroblast growth factor
DMEM: Dulbecco's modified Eagle's medium
DMMB: dimethylmethylene blue
ECM: extracellular matrix
EDTA: ethylenediaminetetraaceticacid
ESC: embryonic stem cell
FBS: fetal bovine serum
hESC: human embryonic stem cell
hTERT: human telomerase reverse transcriptase
l-Glut: l-glutamine
MM: maintenance medium
MSC: mesenchymal stem cell
NEAA: nonessential amino acids
PBS: phosphate-buffered saline
PChM: prochondrogenic medium
PD: population doubling
qRT-PCR: quantitative reverse transcription–polymerase chain reaction
sGAG: sulfated glycosaminoglycan
TGF: transforming growth factor
